# Biophysical Assessment of Human Aquaporin-7 as a Water and Glycerol Channel in 3T3-L1 Adipocytes

**DOI:** 10.1371/journal.pone.0083442

**Published:** 2013-12-20

**Authors:** Ana Madeira, Marta Camps, Antonio Zorzano, Teresa F. Moura, Graça Soveral

**Affiliations:** 1 Research Institute for Medicines and Pharmaceutical Sciences (iMed.UL), Faculty of Pharmacy, University of Lisbon, Lisbon, Portugal; 2 Departmento de Bioquímica e Biologia Humana, Faculdade de Farmácia, Universidade de Lisboa, Lisboa, Portugal; 3 Institute for Research in Biomedicine (IRB Barcelona), Barcelona, Spain; 4 Departament de Bioquímica i Biologia Molecular, Facultat de Biologia, Universitat de Barcelona, Barcelona, Spain; 5 CIBER de Diabetes y Enfermedades Metabólicas Asociadas (CIBERDEM), Instituto de Salud Carlos III, Madrid, Spain; 6 REQUIMTE, Departamento de Química, Faculdade de Ciências e Tecnologia, Universidade Nova de Lisboa, Caparica, Portugal; Graduate School of Medicine, the University of Tokyo, Japan

## Abstract

The plasma membrane aquaporin-7 (AQP7) has been shown to be expressed in adipose tissue and its role in glycerol release/uptake in adipocytes has been postulated and correlated with obesity onset. However, some studies have contradicted this view. Based on this situation, we have re-assessed the precise localization of AQP7 in adipose tissue and analyzed its function as a water and/or glycerol channel in adipose cells. Fractionation of mice adipose tissue revealed that AQP7 is located in both adipose and stromal vascular fractions. Moreover, AQP7 was the only aquaglyceroporin expressed in adipose tissue and in 3T3-L1 adipocytes. By overexpressing the human AQP7 in 3T3-L1 adipocytes it was possible to ascertain its role as a water and glycerol channel in a gain-of-function scenario. AQP7 expression had no effect in equilibrium cell volume but *AQP7* loss of function correlated with higher triglyceride content. Furthermore it is also reported for the first time a negative correlation between water permeability and the cell non-osmotic volume supporting the observation that AQP7 depleted cells are more prone to lipid accumulation. Additionally, the strong positive correlation between the rates of water and glycerol transport highlights the role of AQP7 as both a water and a glycerol channel and reflects its expression levels in cells. In all, our results clearly document a direct involvement of AQP7 in water and glycerol transport, as well as in triglyceride content in adipocytes.

## Introduction

Aquaporins (AQPs) belong to a highly conserved group of membrane proteins that are involved in the transport of water and small solutes and that play a variety of important physiological roles. The 13 human AQP isoforms (AQP0–12) are differentially expressed in many types of cells and tissues in the body and can be divided into two major groups: those strictly selective for water (orthodox aquaporins), and those that are also permeable to other small solutes including glycerol (aquaglyceroporins). The latter include isoforms AQP3, AQP7, AQP9, and AQP10 [1].

The plasma membrane aquaporin-7 (AQP7) was shown to be expressed in adipose tissue. It is well accepted that obesity results from an increase in size and number of adipose cells primarily due to intracellular lipid accumulation in the form of triacylglycerol. There is evidence pointing towards the role of AQP7 in glycerol release/uptake in adipocytes [2] and a correlation between AQP7 deregulation and the development of obesity has been postulated [3].

Several studies have been attempting to disclose the possible connection between AQP7 and obesity/diabetes. Nevertheless it is still difficult to pinpoint the real impact of AQP7 adipose expression in these disorders. Studies conducted in AQP7 null mice have related the depletion of AQP7 to the development of obesity and adipocyte hypertrophy. There is evidence that AQP7 deficiency leads to glycerol retention within adipose tissue, ultimately leading to acceleration of triglyceride synthesis and accumulation in mice adipocytes [3], [4]. However, in obese *db*+/*db*+ mice *AQP7* mRNA levels and plasma glycerol concentration in the interstitial fluid of adipose tissue were found elevated [2], [5]. Moreover, several expression studies in human adipose tissue, although not always in complete agreement, point to the upregulated *AQP7* expression in visceral fat depots and downregulated *AQP7* expression in subcutaneous fat mass in human obesity and type 2 diabetes disorders [6], [7], [8]. Our opinion is that there is a lack of functional studies of AQP7 within its native/physiological context, the adipocyte. Moreover, AQP7 has been considered a glycerol and water channel based only on three main evidences: expression in *Xenopus oocytes* enhanced water and glycerol permeability [9]; Aqp7-knockout (KO) mice show lower plasma glycerol levels and impaired glycerol release in response to beta3-adrenergic agonist [3] and glycerol permeability is reduced in AQP7-ablated adipocytes [4]. Overall, despite the outcome of AQP7 deficiency in adipocytes has been generally studied, the consequence of its overexpression has never been analyzed. On the other hand, the undetectable AQP7 labeling in adipocyte membranes does not support the appointed role for AQP7 in glycerol transport in adipocytes [10].

In view to this, our efforts were firstly directed towards unraveling the precise location of AQP7 within the adipose tissue. Furthermore, we aimed at characterizing AQP7 channel kinetics activity by evaluating glycerol and water permeability in an adipose stable cell line and to investigate the adipocyte overall size and volume dependency on AQP7 expression. Our work approach, on one hand, comprehended the loss of function situation, by knocking down AQP7 in 3T3-L1 adipocytes so as to characterize the transport properties of the mice protein isoform; on the other hand, having characterized the mice system, we assembled the gain of function scenario, by overexpressing the human AQP7 in 3T3-L1 adipocytes, aiming at the functional characterization of the human isoform.

## Materials and Methods

### Ethics Statement

The protocol was conducted according to the European Guidelines for the Care and Use of Laboratory Animals (Directive 86/609) and approved by the University of Barcelona Committee on Animal Care.

### 3T3-L1 cell culture

3T3-L1 fibroblasts (CCL 92.1; American Type Culture Collection, Manassas, VA) were grown to confluence and induced to differentiate into adipocytes essentially as described [11]. Fully mature adipocytes were used 10–15 days after initiation of differentiation.

### Isolation of Adipocytes and Stroma vascular fraction (SVF) from white adipose tissue

Adipocytes and SVF cells were isolated from freshly excised mice visceral white adipose tissue as previously described [12]. Briefly, C57BL/6 mice were anesthetized with isoflurane and visceral white adipose tissue was removed, washed with 0.9% NaCl and digested. Thirty mice were used for each experiment. The minced pieces of white adipose tissue were resuspended in oxygenated incubation buffer containing 154 mM NaCl, 154 mM MgSO_4_, 110 mM CaCl_2_, 154 mM KCl, 200 mM NaH_2_PO4.2H2O, 200 mM NaHPO_4_.2H_2_O and 1.5 M Hepes, 2 mM sodium pyruvate, 3.5% (m/v) BSA and 0,66 mg/mL collagenase, and digested in a shaking water bath at 37°C for 1 hour. The digested pieces were filtered through a nylon mesh to remove tissue remnants and centrifuged at 2300×g for 5 minutes. After washing twice the floating adipocytes and the pelleted SVF with incubation buffer, these fractions were frozen in liquid nitrogen and used immediately for RNA isolation.

### RNA extraction and RT-PCR

Total RNA was extracted with RNeasy Mini Kit (Qiagen) and treated with DNase I (Invitrogen) to remove any trace of genomic DNA. The RNA was kept at −80°C until further assay. RNA concentration was determined by spectrophotometry at an absorvance of 260 nm and RNA purity confirmed by the OD_260_/OD_280_ absorption ratio. Complimentary DNA (cDNA) was obtained using 1 µg total RNA and the retrotranscription reaction was carried out with SuperScript® II RT (Invitrogen) and oligo dT (Roche). The quantification of the PCR products was accomplished either by measuring the fluorescence of specific probes for each target sequence (Taqman ® pre-designed gene expression assays, Applied Biosystems) or by measuring fluorescence from the progressive binding of SYBR green I dye to double stranded DNA. Amplification and detection of specific products were performed with the 7500 Real-Time PCR System (Applied Biosystems) following the manufacturer's protocol. The relative quantification value of PCR transcripts was calculated either using the comparative Ct method (manufacture's protocol) or the standard curve method [13] with normalization to aRP (acidic ribosomal protein) or eEF2 (eukaryotic translation elongation factor 2) as endogenous controls. The set of specific primers and TaqMan ® pre-designed gene expression assays were as follows: AQP3 (Mm01208559_m1); AQP7 (Mm00431839_m1); AQP9 (Mm00508094_m1); aRP (probe Mm01974474_gH); GLUT4 (5′-ACTTCATTGTCGGCATGGGT-3′ and 5′-AGATCTGGTCAAACGTCCGG-3′); Perilipin (5′-TGCTGGATGGAGACCTC-3′ and 5′-ACCGGCTCCATGCTCCA-3′); aP2 (fatty acid binding protein 4) (5′-TTCGATGAAATCACCGCAGA-3′ and 5′-GGTCGACTTTCCATCCCACTT-3′); HSL (Hormone sensitive lipase) (5′-GGCTTACTGGGCACAGATACCT-3′ and 5′-CTGAAGGCTCTGAGTTGCTCAA-3′); SOX9 (5′- CGTTCTTCACCGACTTCCTC-3′ and 5′- AGGAAGCTGGCAGACCAGTA-3′); hAQP7 (5′AGTTCCTGGGCTCCTTCCTG 3′ and 5′GAACCAAGGCCGAATACCATC 3′) and eEF2 (5′ GCTTCCCTGTTCACCTCTGACTCTG 3′ and 5′ CCGGATGTTGGCTTTCTTGTCC 3′).

### cDNA and shRNA Constructs

Human AQP7 was subcloned from the pENTR™221 vector (Invitrogen) into the lentiviral expression vector pWPI-DEST (Adaptation by Trono Lab) using the recombination Gateway® Technology according to the manufacturer's instructions (LR clonase, Invitrogen). pWPI-DEST is a bicistronic vector that allows for simultaneous expression of a transgene and EGFP (Enhanced Green fluorescence protein) marker to facilitate tracking of transduced cells. The obtained clones were verified by DNA sequencing (BigDye sequencing kit, Applied Biosystems).

For mice AQP7 silencing, 5 different target shRNA constructs (MISSION® shRNA Mouse Gene Family Set, Sigma-Aldrich) inserted within the lentiviral plasmid vector pLKO.1-puro were used [14]. The effective target sequences of the sense shRNA were as follows: 5′ – GCCTTGTGTATGCTAGGTAAT – 3′ (Clone ID TRCN0000102160); 5′ – GCAGAGTTCCTGAGTACCTAT – 3′ (Clone ID TRCN0000102161). shRNA lentiviral non-target control plasmid (MISSION pLKO.1-puro Control Transduction Particles, Catalog Number SHC001V, Sigma-Aldrich) was used as a control. The pLKO.1–Puro vector contains a puromycin resistance marker for selection of inserts in successfully infected cells.

### Lentivirus production and infection of 3T3-L1 preadipocytes

To generate the lentivirus, shRNA or cDNA lentiviral expression constructs were co-transfected into Human kidney 293T cells with pCMVR8.74 (Addgene plasmid 22036) and pMD2G (Addgene plasmid 22036) using the PEI (polyethilenimine) method [15]. The HIV derived constructs (pCMVR8.74 helper packaging vector and pMD2G vector encoding for envelop protein) were kindly provided by Dr. D. Trono from the *Ecole polytechnique Federale de Lausanne* (Switzerland). Culture medium containing lentivirus was harvested at 48 and 72 h post-transfection, and filtered through 0.45 µm filters to remove cell debris. Recombinant lentiviruses were then purified by ultracentrifugation in a 20% sucrose cushion.

3T3-L1 preadipocytes grown on 6 well plates (1×10^4^ cells/mL) were infected and amplified for 3 days, after which selection with 3 µg/mL puromycin (Sigma) was initiated (for the shRNA constructs) or sorting in a MoFlo flow cytometer (Dako Diagnostics SA, Barcelona, Spain; Submmit version 3.1 software) was performed to detect and collect GFP-positive cells.

### Measurement of triglyceride content

The quantification of total triglyceride content from adipocytes was performed by obtaining total homogenates using HES buffer (0.25 M Sucrose, 2 mM EGTA, 20 mM HEPES, pH = 7.4). The homogenate was used for total triacylglyceride measurement using a commercially available kit (Biosystems Triglyceride assay kit) and following the manufacturer's instructions. The homogenate was also used for protein measurement using the Pierce® BCA Protein Assay Reagent (Thermo Scientific) and the final result was expressed relative to protein levels.

### Permeability assays

Water (osmotic permeability coefficient, *P*_f_) and glycerol (glycerol permeability coefficient, *P*_gly_) permeabilities were measured in individual adherent cells on a coverslip. Briefly, 3T3-L1 adipocytes were loaded with 5 µM calcein acetoxymethyl ester (calcein-AM) (Sigma® Aldrich) (a volume sensitive fluorescence probe) for 90 min at 37°C in 5% CO_2_/95% air. The coverslips with the adhered cells were mounted in a closed perfusion chamber (Warner Instruments, Hamden, USA) on the stage of a Zeiss Axiovert 200 inverted microscope. Fluorescence was excited at wavelength 495/10 nm and the emission fluorescence was collected with a 535/25 nm bandpass filter coupled with a 515 nm dichroic beam splitter. Images were captured using a ×40/1.6 epifluorescence oil immersion objective and a digital camera (CoolSNAP EZ, Photometrics, USA) and were recorded by the Metafluor Software (Molecular Devices, USA).

For the *P*_f_ assessment, cells were perfused with HEPES (135 mM NaCl, 5 mM KCl, 2.5 mM CaCl_2_, 1.2 mM MgCl_2_, 10 mM Glucose, 5 mM Hepes, pH 7.4, initial osmolarity (osmout)_o_ = 300 mosM) for 60 s, after which 300 mM mannitol (non-diffusible solute) was added, being achieved an external osmolarity (osmout)_∞_ = 600 mosM and thus a tonicity of the osmotic shock (Λ) of 2 (Λ is defined as the ratio between final and initial media osmolarities, Λ  = (osmout)_∞_/(osmout)_o_).

In order to evaluate glycerol permeability *P*_gly_, two protocols using glycerol as a diffusible solute were executed (with and without osmotic shocks). On the 1^st^ Protocol, cells equilibrated in isotonic 300 mM Hepes solution were subjected to hypertonic shocks by the addition of 300 mM glycerol (a permeable solute) being achieved an external osmolarity of 600 mosM and thus a tonicity of the osmotic shock (Λ) of 2. On the 2^nd^ Protocol, the substitution protocol, cells equilibrated for 60 s in isotonic solution containing 200 mM mannitol (200 mM mannitol, 35 mM NaCl, 5 mM KCl, 2.5 mM CaCl_2_, 1.2 mM MgCl_2_, 10 mM Glucose, 5 mM Hepes, pH 7.4, (osmout)_o_ = 300 mosM) were suddenly exposed to the same perfusate where mannitol was replaced by 200 mM glycerol. Under these conditions, no osmotic shock was applied (Λ = 1).

### Osmotic and Glycerol Permeability Coefficients

Permeability coefficients *P*_f_ and *P*_gly_ were evaluated from the measured time dependent volume changes, v_rel_ = V/V_o_, obtained by adding mannitol (*P*_f_ estimation) or glycerol to the external media achieving an osmotic challenge of Λ = 2, or by substituting the impermeant solute mannitol by glycerol (Λ = 1) (estimation of both *P*_f_ and *P*_gly_).

*P*_f_ was evaluated from the mannitol osmotic shock, as mannitol is considered to be impermeant, and this value was used as a first approximation when evaluating *P*_gly_ (using the above mentioned protocols). The relative non-osmotic volume β = V_Nosm_/V_o_ was considered in all calculations.

### Model 1: Water and Solute fluxes induce cell volume changes

When cells are subjected to an osmotic challenge with a non-diffusible (ND) or diffusible (S) solute, the resulting changes in cell volume depend not only on the water permeability but also on the solute permeability. Considering that there are no convective fluxes (diffusible solute reflection coefficient σ_S_ = 1) and no hydrostatic pressure differences between the intra and extra cellular compartments, the simplified flux equations for water (*J_v_*) and diffusible solute (*J_S_*) from the inner to the outer compartment are:
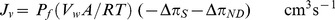
(1)

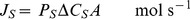
(2)where Δπ_S,ND_ = RT[C_S.ND_in_−C_S,ND_out_] are the osmotic pressure gradients due to the concentration gradients of diffusible (ΔC_S_) and non-diffusible (ΔC_ND_) solutes, V_w_ is the partial molar volume of water (18 cm^3^/mol), A is the membrane surface area, R is the gas constant and T is the absolute temperature.

Upon an osmotic challenge, a volume change induced by water flux occurs and the relative volume (v_rel_ = V/V_o_) change can be directly evaluated by dv_rel_/dt = −J_v_/V_o_. However, in cells, only the osmotic volume (V_osm_ = V−V_Nosm_) and its relative counterpart v_osm_ = V_osm_/V_o_ are altered, while the non-osmotic volume V_Nosm_ and its relative counterpart β = V_Nosm_/V_o_ are constant for each cell population. As β is constant, dv_rel_/dt = dv_osm_/dt. During the osmotic shock, when the relative volumes (v_rel_ and v_osm_) are changing, the intracellular concentrations of S and ND are also changing and can be calculated by their intracellular quantities Q_S,ND_in_ and V_osm_, C_S,ND_in_ = Q_S,ND_in_/(V_osm_). While Q_ND_in_ is constant and equal to Q_ND_in_ = (V_osm_)_o_.(osm_out_)_o_, Q_S_in_ changes and can be evaluated from J_S_, dQ_S_in_/dt = −J_S_, knowing its initial condition (Q_S,ND_in_)_o_ = 0.

For this model 1, if the only gradient to be considered is from non-diffusible species Δπ_ND_ (e.g. mannitol), the parameters to estimate from each experimental trace are reduced to two, *P*_f_ and β. If, on the other hand, there is also a diffusible solute gradient Δπ_S_ (e. g. glycerol) and assuming σ_S_≈1, the parameters to be estimated are the permeability coefficients *P*_f_ and *P*_gly_ and β. This assumption reduces the number of parameters to be evaluated from each experimental trace and implies a delay in the cell response (volume change) in the substitution protocol immediately after solution replacement. However if σ_S_ <1, in equation 1 the osmotic gradient due to solute S would be σ_S_Δπ_S_ and in equation 2 the convective term would have to be considered J_v_(1−σ_S_)C_S_av_, where C_S_av_ is the average concentration of S inside the pore. In this case the number of parameters to be estimated are four (*P*_f_, *P*_gly_, β and σ_S_).

### Model 2: Cells in osmotic equilibrium, volume changes instantaneously following glycerol uptake

For the substitution protocol (2^nd^ Protocol), *P*_gly_ was estimated using two different approaches, the one described above where cell volume changes are due to water and glycerol fluxes (dependent on *P*_f_ and *P*_gly_), and a second approach that considers cells always in osmotic equilibrium (by assuming *P*_f_>>>*P*_gly_), making the model independent of *P*_f_ and thus reducing to two the number of parameters to evaluate (*P*_gly_ and β). The equations are similar with the exception that the equation for J_v_ is not used and changes in V (or v_rel_) are now calculated directly from the changes in V_osm_ (or v_osm_) that results from changes in C_S,ND_in_. In order to evaluate this term, the osmotic equilibrium assumption (Δπ_S_+Δπ_ND_ = 0) was used, C_S,ND_in_ = C_S,ND_out_−(C_S_in_−C_S_out_). Knowing that Q_ND_in_ is always constant, V_osm_ = Q_ND_in_/C_S,ND_in_ and v_rel_ =  v_osm_+β.

Parameters (*P_f_*, *P*_gly_ and β) were evaluated by numerically integrating and curve fitting the time dependent v_rel_ data, using the Berkeley Madonna software (http://www.berkeleymadonna.com/).

### Cell volumes and fluorescence output

A linear relationship between relative changes in cell osmotic volume (V−β)/(V_o_−β) (thus of V/V_o_) and calcein fluorescence intensity (F/F_o_) was previously validated [16].Taking this into account, the cell fluorescence traces F/F_o_ were converted into (V/V_o_) after subtracting the bleaching given by the initial fluorescence decay before the mannitol osmotic shock. F_o_ was calculated in each signal as the averaged initial values of fluorescence prior to the osmotic challenge.

Cell volume V was measured at selected time points from 2D images obtained during the permeability assay protocols (V_o_ is the initial volume prior to the osmotic challenge). For each coverslip with adhered cells, 6 or 18 pictures with 10–13 cells each were analyzed for selected time points for the water and the glycerol permeability studies, respectively. For each experimental condition four coverslips from two different cell platings were assayed, making an average of 40–50 cells analyzed per condition. The cross sectional area of calcein-AM loaded cells was measured using the Image J software and cells were assumed to have a spherical shape for volume calculations.

### Statistical Analysis

The results were expressed as mean ± SD of *n* individual experiments. Statistical analysis between groups was performed by one-way ANOVA followed by Tukey multiple comparisons test or unpaired *t*-test. *P* values <0.05 were considered statistical significant. Pearson's correlation coefficients were calculated to establish linear relationships between the *P*_f_, *P*_gly_ and the non-osmotic cell volume. Statistical analyses were performed using the Graph Prism software (GraphPad Software).

## Results

### AQP7 is both present in isolated adipocytes and in stromal vascular fraction

**Figure 1:**
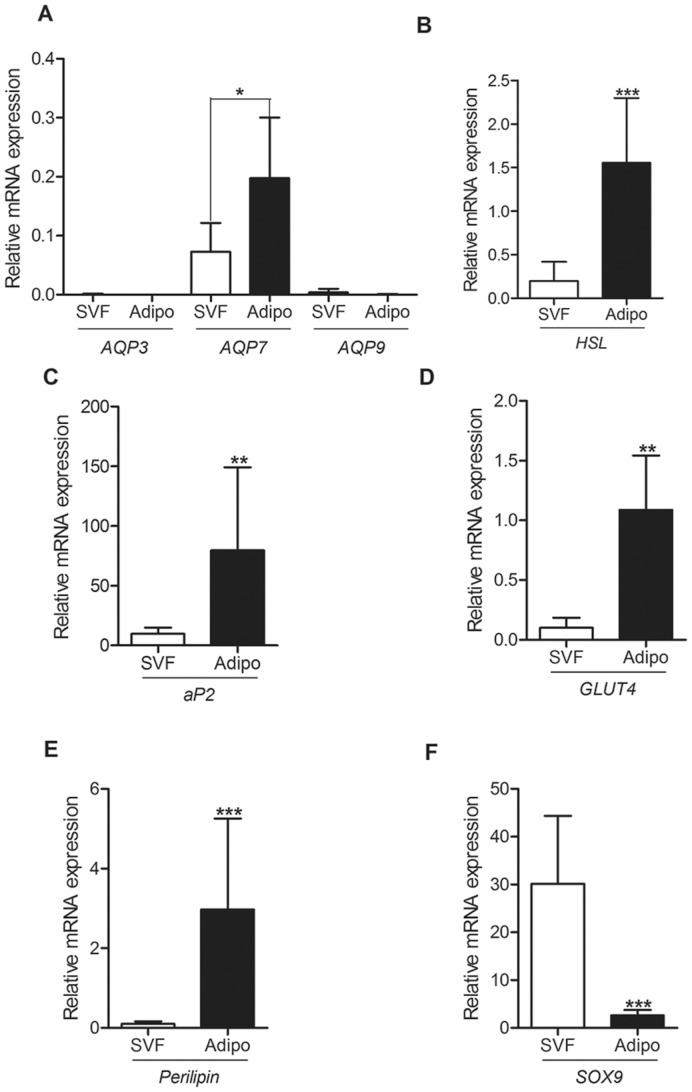
*AQP7* expression is higher in isolated mice adipocytes than in the stromal vascular fraction (SVF) of adipose tissue. A- *AQP3*, *AQP7* and *AQP9* mRNA expression in isolated mice adipocytes (black bars) and SVF (white bars). B–F - mRNA levels of specific markers of mature adipocytes (*aP2*, *GLUT4*, *HSL* and *Perilipin*) and of the capillary endothelia of adipose tissue (*SOX9*). *eEF2* (eukaryotic translation elongation factor 2) mRNA levels were used as reference genes. Data represent mean ± SD derived from five independent experiments. Statistically significant differences detected by Student's t are indicated by ** *P*<0.01; *** *P*<0.001.

Several studies have reported the expression of AQP7 in adipose tissue and have shown evidence for AQP7 involvement in glycerol release from adipocytes [4], [17]. However, Skowronski and co-workers (2007) when performing immunohistochemical and immunoelectron microscopy analysis in the mice white adipose tissue, detected AQP7 in the stromal vascular fraction (SVF) but not in the adipocytes membranes. In order to clarify these apparent contradictions we obtained separate fractions of adipocytes and capillary endothelial from mice white adipose tissue and screened both fractions for the presence of all aquaglyceroporins expressed in mice (*AQP3*, *AQP7* and *AQP9*). As shown in Figure 1A, no expression of AQP3 or AQP9 was detected, while *AQP7* was present in both fractions, being approximately 3 times more expressed in the isolated adipocytes than in the SVF. Expression studies carried out with specific adipocyte and endothelial cell markers (*aP2*, *GLUT4*, *HSL*, Perilipin and *SOX 9*, further detailed in Table S1, Table S2 and Figure S1) ascertained for the purity of each fraction (Figure 1B–F). Similar results had been previously obtained in fractionated samples of human adipose tissue [8], [18], suggesting an involvement of AQP7 in transport specifically across the endothelium barrier of white adipose tissue.

### Aquaglyceroporin expression levels in 3T3-L1 adipocytes

In order to evaluate the expression level of the three aquaglyceroporins already identified in mice, we screened for the presence of *AQP3*, *AQP7* and *AQP9* in 3T3-L1 fibroblasts and adipocytes. Semi-quantitative real time RT-PCR experiments revealed that *AQP7* is the only aquaglyceroporin expressed in mature 3T3-L1 adipocytes, with negligible expression in fibroblasts (Table 1), which is in agreement with our previous results for mice adipose tissue (Fig. 1A). This finding confirms the results already reported for mature adipocytes [2] as well as for mice [17] and human [8] adipose tissue, where *AQP7* was the only aquaglyceroporin detected.

**Table 1:** Aquaglyceroporin expression levels (arbitrary units) in 3T3-L1 cell lines.

Cell line	Gene	Fibroblasts	Adipocytes
*3T3-L1*	*mice AQP3*	nd	nd
	*mice AQP7*	0.002 (±0.003)	1.707 (±0.816)^a^
	*mice AQP9*	0.063 (±0.140)	nd
*Scramble Control*	*mice AQP7*	-	0.011 (±0.004)
*AQP7-shRNA*	*mice AQP7*	-	0.002 (±0.001)^b^
*GFP*	*human AQP7*	nd	-
*hAQP7*	*human AQP7*	1.225 (±0.285)	-

Semi-quantitative real-time PCR analysis *AQP3*, *AQP7* and *AQP9* expression in 3T3-L1 cells, 3T3-L1 cells transduced with scramble shRNA (Scramble control), transduced with a lentivirus expressing short-hairpin RNA that targets *AQP7* (*AQP7-shRNA*), or transduced with a lentivirus encoding GFP (GFP) or human *AQP7* plus GFP (h*AQP7*).

Each value represents the mean ±SD of the ratio between each transcript and *aRP* (n = 5). ^a^ *P*<0.01 vs Fibroblasts; ^b^ *P*<0.001 vs. Scramble Control; nd, not detected.

### AQP7 gain-of-function and loss-of-function

Previous studies have shown that AQP7 is an adipogenic specific marker and that its expression in 3T3-L1 pre-adipocytes increases in parallel with glycerol release activity during differentiation [2]. In order to establish the direct role between the levels of AQP7 expression and the adipocyte membrane permeability to glycerol and water, we obtained 3T3-L1 stable cell lines, in which *AQP7* levels were either reduced (mice *AQP7* knockdown phenotype, *AQP7*-shRNA) or augmented (overexpressing phenotype for human *AQP7*, h*AQP7*) (Table 1). The knockdown cell line showed an 85% reduction of *AQP7* transcript level; as for the overexpression line, an enhanced expression level of *AQP7* was detected.

### Cell volume of adipocytes with different levels of AQP7 expression

**Figure 2:**
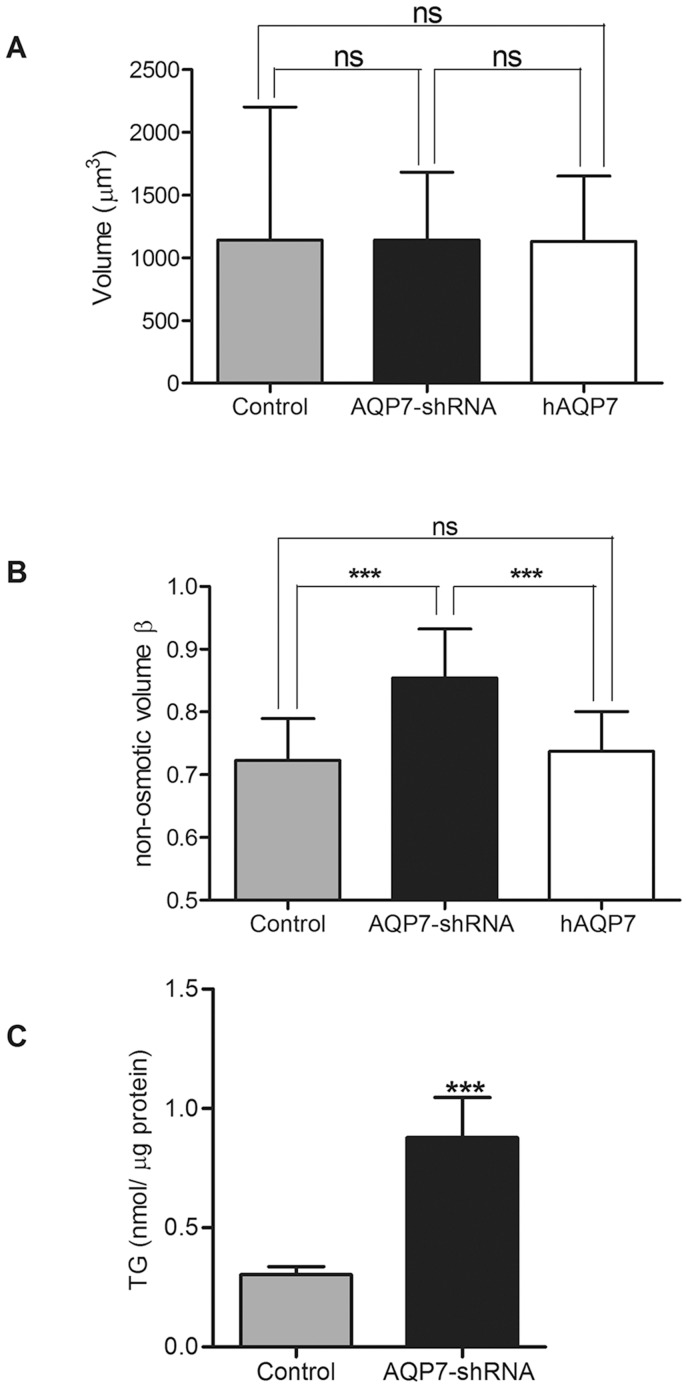
*AQP7* expression has no effect in equilibrium cell volume but depletion of *AQP7* correlates with higher non-osmotic volume and triglyceride content. A- Equilibrium cell volumes of 3T3-L1 adipocytes infected with scramble shRNA (Scramble control), *AQP7* knockdown adipocytes (AQP7-shRNA) and human *AQP7* overexpressing adipocytes (hAQP7). Bars show mean ±SD from 40–50 cells analyzed on 4 coverslips in 2 cell platings. B- Non-osmotic volumes β of 3T3-L1 adipocytes infected with scramble shRNA (Scramble control), *AQP7* knockdown adipocytes (*AQP7*-shRNA) and human *AQP7* overexpressing adipocytes (h*AQP7*). Bars show mean ±SD from 40–50 cells analyzed on 4 coverslips in 2 cell platings. C- Intracellular triglyceride (TG) content in 3T3-L1 adipocytes infected with scramble shRNA (Control) and in *AQP7* knockdown adipocytes (*AQP7*-shRNA). Bars show the mean ±SD of 6 separate measurements. Significance levels: ns, not significant, *P*>0.05; * *P*<0.05; ** *P*<0.01; *** *P*<0.001, given either by one-way ANOVA followed by Tukey's *post-hoc* test or by Student's *t* test.

It has been hypothesized that an increase in density of functional AQP7 glycerol channels in the plasma membrane could be relevant for glycerol release or accumulation, therefore having an outcome on lipid droplet content and cell volume. Thus, we evaluated the cell volumes of 3T3-L1 cell lines expressing differential levels of *AQP7* by measuring the cross-sectional area of calcein loaded cells in osmotic equilibrium (V_o_) and assuming a spherical shape. In Figure 2A it is observed that there was no correlation between *AQP7* expression level and the total cell volume.

We also assessed the relative non-osmotic volume (β), which represents the fraction of the cell that is osmotically unresponsive. In adipocytes, besides the intracellular organelles, the increase in osmotically inactive portion of these cells is greatly due to the existence of large lipid droplets that occupy most of cells' cytoplasm. By measuring the equilibrium cell volume before and after osmotic challenges with mannitol in calcein loaded adipocytes, it was possible to assess the osmotically inactive volume for the different cell lines.

It was observed that in control 3T3-L1 adipocytes, around 72% of the total cell volume was osmotically inactive, meaning that only a small portion of the cytoplasm respond to osmotic challenges (Figure 2B). No significant differences regarding the non-osmotic volumes were found for control and *AQP7* overexpressing 3T3-L1 adipocytes. However, depletion of *AQP7* correlates with an increase in the non-osmotic volume (β), since in *AQP7* knockdown adipocytes around 86% of the total volume was osmotically inactive, 14% more than in control cells (*P*<0.001). These results are in agreement with the elevated triglyceride content measured in adipocytes depleted of AQP7 [3], [4], therefore explaining an increase in the lipid droplet size. To ascertain that the assessed β differences were due to increased triglyceride storage depots, we compared the triglyceride content of the two cell lines that gave different β values, the control and *AQP7* knockdown adipocytes, aiming to correlate the triglyceride accumulation and the non-osmotic volume of the cell. As depicted in Figure 2C, the total amount of triglycerides was nearly three times higher in *AQP7* depleted adipocytes, confirming our initial hypothesis.

### Functional role of AQP7 on water and glycerol permeability

Plasma membrane permeability to water and glycerol were evaluated in 3T3-L1 adipocytes expressing different levels of *AQP7*: control 3T3-L1 adipocytes, adipocytes silenced for *AQP7* and adipose cells overexpressing human *AQP7*.

**Figure 3:**
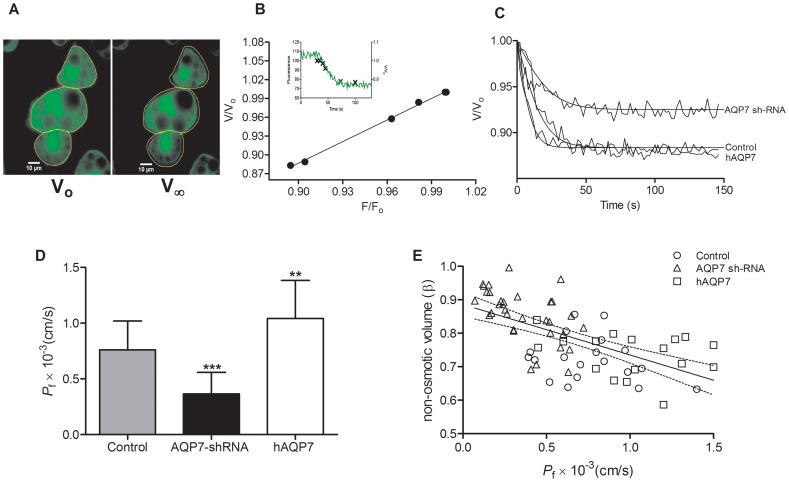
Functional assessment of *AQP7* water transport. Water permeability was assayed by epifluorescence microscopy in adipocytes infected with scramble shRNA (Scramble control), *AQP7* knockdown adipocytes (*AQP7*-shRNA) and human *AQP7* overexpressing adipocytes (h*AQP7*). **A** – Representative illustration of calcein loaded cells with initial equilibrium volume V_o_ (left panel) and final equilibrium volume V_∞_ (right panel) after an osmotic challenge of tonicity two with mannitol. **B** – Relationship between changes in cell volume (V/V_0_) and fluorescence intensity (F/F_0_) (n = 5 cells). Individual calibration was performed for each fluorescence mannitol experiment. Inset shows a typical fluorescence (F) trace together with measured cell volumes (V/Vo) along the experiment. **C** - Representative time course of the relative cell volume change V/V_o_ for 3T3-L1 adipocytes expressing different levels of *AQP7* after an osmotic shock of tonicity two with mannitol. **D** - Osmotic water permeability coefficient (*P*_f_). **E** – β dependence on *P*_f_. The linear fit and the 95% confidence band are shown. Bars show mean ±SD from 40–50 cells analyzed on 4 coverslips in 2 cell platings. Significance levels: ns, not significant, *P*>0.05; * *P*<0.05; ** *P*<0.01; *** *P*<0.001, given by one-way ANOVA followed by Tukey's post-hoc test.

The osmotic water permeability coefficient *P*_f_ was determined by computing the time course of cell volume change of equilibrated cells subjected to a hyperosmotic challenge by the addition of a non-diffusible solute (mannitol) (Figure 3A). This figure depicts cells in initial and final equilibrium stages with their initial and final volumes V_o_ and V_∞_. A linear relationship between V/V_o_ and F/F_o_ was found for these calcein loaded cells (Figure 3B) allowing the calibration of the fluorescence output after the mannitol osmotic challenge. Furthermore, this linear correlation suggests that the overestimation of cellular volume V when considering a spherical rather than a disc-like shape of adherent cells might be disregarded for relative volume changes V/V_o_.

Figure 3C shows the time course of the relative cell volume change (V/V_o_) for 3T3-L1 adipocytes expressing different levels of *AQP7* subjected to an osmotic shock with mannitol, inducing water outflow and cell shrinkage. It can be observed that adipocytes depleted of *AQP7* show decreased signal amplitudes when compared with control and *AQP7* overexpressing adipocytes, pointing to reduced volume change for the same osmotic challenge. On the contrary, control and *AQP7* overexpressing adipocytes showed equivalent final volume changes. These observations are in agreement with our data from the relative non-osmotic volume (β) described above (Figure 2B), since the higher non-osmotic volume is preventing *AQP7* knockdown adipocytes to reach the same shrinkage minimum volume as the other cell lines.

In addition, in Figure 3C a slower time course of volume change is depicted for *AQP7* knockdown adipocytes. The calculated *P*_f_ values are represented in Figure 3D, where the *P*_f_ value for adipocytes overexpressing *AQP7* ((1.04±0.34)×10^−3^ cm s^−1^) was 1.36 fold higher than the control ((0.76±0.26)×10^−3^ cm s^−1^). Comparing with the control, a reduction of more than 50% was obtained for the *P*_f_ value in *AQP7* knockdown adipocytes ((0.36±0.19)×10^−3^ cm s^−1^). Our results suggest that AQP7 plays a relevant role controlling water permeability in 3T3-L1 adipose cells.

Figure 3E shows a negative correlation between *P*_f_ and non-osmotic volume β (*P*<0.0001, *r* = −0.6132). The data points for the knockdown are distributed among the lower *P*_f_ range with correspondent higher β values, followed by the control and the *AQP7* overexpression cells, reinforcing the idea that AQP7 depletion may contribute to lipid accumulation in adipocytes.

**Figure 4:**
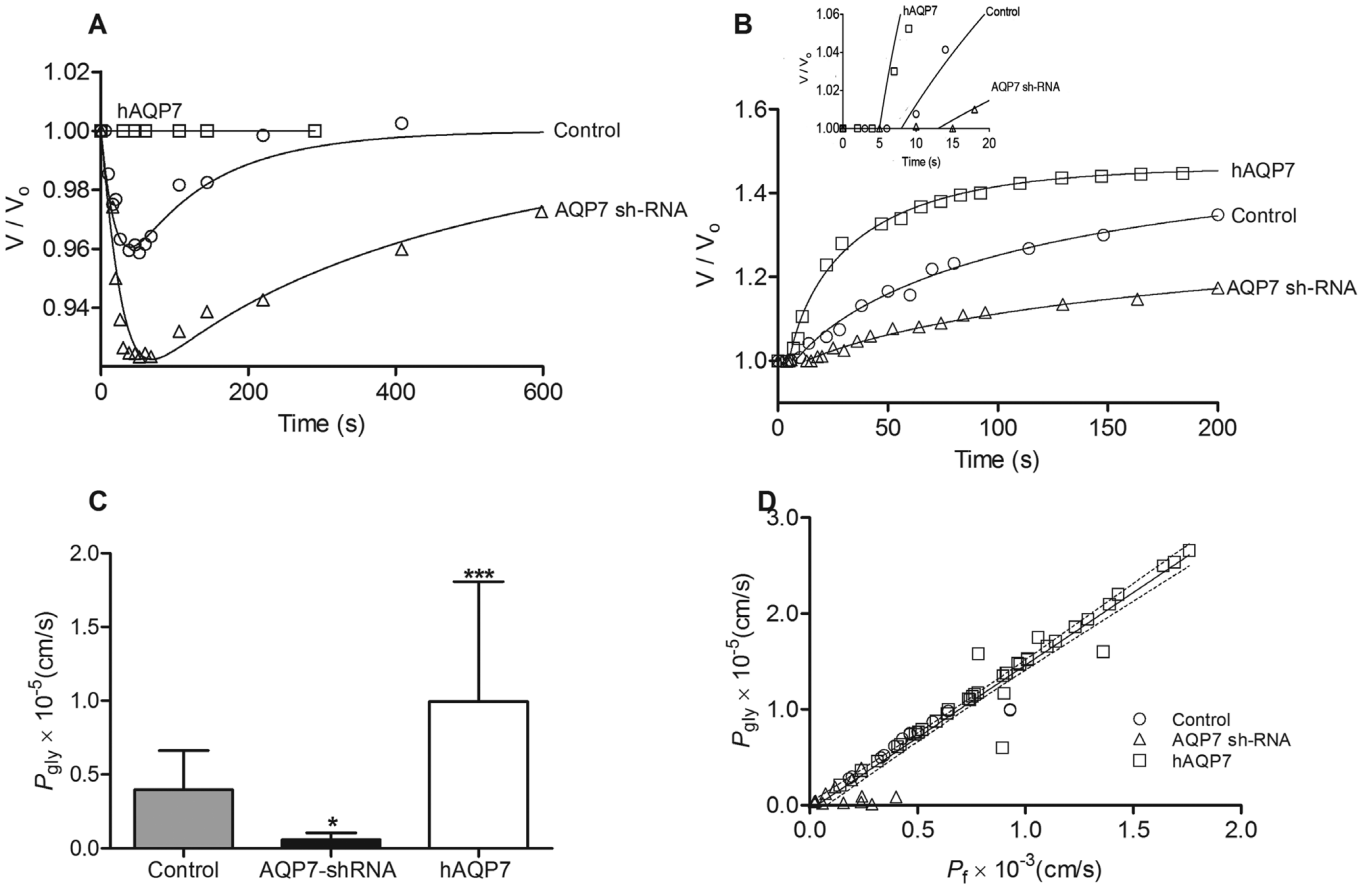
Functional assessment of *AQP7* glycerol transport. Glycerol permeability was assayed by epifluorescence microscopy in 3T3-L1 adipocytes infected with scramble shRNA (Control), *AQP7* knockdown adipocytes (*AQP7*-shRNA) and in human *AQP7* overexpressing adipocytes (h*AQP7*). **A** - Representative time course of the relative cell volume change V/V_o_ for 3T3-L1 adipocytes expressing different levels of *AQP7* but presenting equivalent non-osmotic volumes (β) after an osmotic shock of tonicity two with glycerol. **B** - Representative time course of the relative cell volume change V/V_o_. Cells equilibrated in an isotonic solution containing mannitol were switched to the same solution where mannitol was replaced by glycerol. All data points were from cells presenting equivalent non-osmotic volumes (β). The inset extends the time scale, showing the first 20 seconds of relative cell volume measurements. **C** - Glycerol permeability coefficient (*P*_gly_). **D** - *P_gly_* dependence on *P*_f_. The linear fit and the 95% confidence band are shown. Bars show mean ± SD from 40–50 cells analyzed on 4 coverslips in 2 cell platings. Significance levels: ns, not significant, *P*>0.05; * *P*<0.05; ** *P*<0.01; *** *P*<0.001, given by one-way ANOVA followed by Tukey's post-hoc test.

In order to assess glycerol permeability (*P*_gly_) the measured relative cell volumes during the glycerol experiments were used in the calculations rather than the calibrated fluorescence traces as, for longer experimental protocols the observed fluorescence drifts turned the calibration difficult to perform. We started by monitoring cell volume changes succeeding a glycerol osmotic shock, 1^st^ Protocol (Figure 4A). In this experimental setting, after the perturbation was applied, both water and glycerol fluxes phenomena were concomitantly taking place. In fact, Figure 4A shows that in both control and *AQP7* depleted adipocytes, after the initial fast cell shrinkage due to water outflow, glycerol influx in response to its chemical gradient was followed by water influx, with subsequent cell re-swelling. Given that *P*_f_ is higher than *P*_gly_ an initial cell volume reduction was observed, but since glycerol is permeable, once it enters the cells following its concentration gradient, water fluxes are inverted with consequent cell volume increase. When comparing *AQP7* depleted and control cells, the former presented a slower re-swelling rate and did not recover their initial volume during the experimental timespan. Moreover, when measuring cell volumes for the AQP7 overexpressing cells, no detectable changes were usually observed in most experiments, in contradiction with what was expected. As these cells must have higher permeability values for *P*_f_ and *P*_gly_ with *P*_f_>*P*_gly_, it is expected to find at least a small volume change followed by a rapid recover of the original volumes. The fact that this was not observed may be due to the shortcoming of measuring cell volumes from a 2D image of disk-like shape adherent cells, unable to discriminate very small changes (below 2%) in cell volume. Although with this protocol no values of *P*_gly_ could be obtained, the data suggests an increase in glycerol influx that partially counterbalances the expected water outflow in response to the imposed osmotic pressure gradient, thus making the volume changes very small and difficult to detect. These data clearly illustrate the involvement of AQP7 in glycerol transport.

In order to surpass the difficulty posed by the 1^st^ Protocol and evaluate the *P*_gly_ for *AQP7* overexpressing cells, the substitution protocol was used. *P*_gly_ was estimated by computing the time course of cell volume change in cells equilibrated in an isotonic solution containing mannitol and switched to the same solution where mannitol was replaced by glycerol (Figure 4B). When analyzing the relative cell volume change (V/V_o_) obtained for cells with equivalent non-osmotic volumes and subjected to the same glycerol challenge, it was observed a small delay in the volume response (≈5–7 s for overexpression, 10–15 s for control and 15–30 s for AQP7 knockdown cells, Figure 4B inset). After this short delay, an increase in cell volume was observed. This behavior is consistent with the influx of glycerol in response to its chemical gradient, followed by water influx with subsequent cell swelling. However, it is also observed that the rate of swelling in glycerol solution was related to the level of *AQP7* expression: the higher the expression, the higher the rates of cell volume change. In fact, by the end of the experimental protocol (200 s), control and *AQP7* knockdown adipocytes had reached, respectively, less than 80% and 40% of the final volume already attained by the *AQP7* overexpressing adipocytes.

Figure 4C shows the *P*_gly_ values evaluated from the substitution protocol using the osmotic equilibrium model (model 2). *P*_gly_ of *AQP7* overexpressing cells ((9.93±0.81)×10^−6^ cm s^−1^) was 2.5 and 17 times higher than control ((3.98±0.27)×10^−6^ cm s^−1^) and *AQP7* knockdown ((0.59±0.46)×10^−6^ cm s^−1^) adipocytes, respectively. For the control and depleted cells, using any of the above protocols (with or without osmotic shock), the estimated *P*_gly_ values with model 1 (σ_S_ = 1) were equivalent to the represented on Figure 4C where the model 2 was used, showing that the results were independent of both experimental protocol and model of analysis. For the *AQP7* overexpressing adipocytes the *P*_gly_ values were slightly (1.3 times) higher than the ones computed from the osmotic equilibrium model (model 2).

Moreover, when estimating *P*_gly_ values using model 1 and assuming a value of σ_S_ equal to 1, the *P*_gly_ values were slightly over-estimated for the different *AQP7* expressing adipocytes. By fitting the v_rel_ data for all the cells and considering σ_S_ values ranging from 0.5 to 1, the average estimated *P*_gly_ decreased less than 10% for the lower σ_S_. Therefore, the value of σ_S_ does not greatly affect the differences observed in *P*_gly_ for the different *AQP7* expressing adipocytes (Figure 4C) confirming the depicted data.

Figure 4D shows the strong positive correlation between *P*_gly_ and the correspondent *P*_f_ values obtained with the substitution protocol and analyzed with model 1 (P<0.0001, r = 0.9685). The lower *P*_gly_ values for the knockdown are distributed over the lower *P*_f_ range, followed by higher *P*_gly_ for the control and even higher *P*_gly_ for *AQP7* overexpression within the higher *P*_f_ range. These results support our conclusion that AQP7 functions both as a water and as a glycerol channel, as silencing and overexpressing *AQP7* decreases and increases, respectively, both water and glycerol permeability. Therefore, the permeability values are a reflection of AQP7 levels of expression.

Altogether these data clearly demonstrate a direct involvement of AQP7 in glycerol and water transport and in lipid content in adipocytes.

## Discussion

The present study provides evidence for simultaneous expression of AQP7 both in adipocytes and in the capillary endothelia of mice adipose tissue. In addition, the detection of higher amounts of *AQP7* transcript in adipocytes indicates the likelihood of a higher AQP7 expression at the protein level in this fraction.

The detection of *AQP7* in the stromal vascular preparations of adipose tissue leads us to hypothesize for the involvement of AQP7 in the paracellular transport of both water and glycerol between the bloodstream and the interstitium of white adipose tissue.

By the means of a non-invasive technique it was possible to gain insight of individual cells' response to the applied solute gradients and ultimately assess the values of *P*_f_ and *P*_gly_, with minimal alterations to cells' environmental and physiological status. Firstly, we were able to ascertain that both mice and human AQP7 when expressed in adipocytes are functional as glycerol channels. The uptake of glycerol, reflected by the estimated *P*_gly_ values, was intimately connected to the AQP7 transcript levels, being greatly impaired with the deletion of AQP7.

Furthermore we also observed a connection between *AQP7* deficiency and an increase in the non-osmotic volume, which was attributed to triglyceride accumulation within the adipocyte. Indeed, an adipocyte volume increase in obesity was associated to AQP7 depletion in knock-out mice [3], [4]; however, in our study, the increased non-osmotic volume was not matched by an accompanying increased cell volume. It is possible that the residual AQP7 in the *AQP7* knockdown cells is sufficient to maintain the minimum glycerol exchanges required to guarantee an unchanged adipocyte size. Moreover, the inability of human *AQP7* overexpression to diminish the adipose cell lipid content (with no changes in the non-osmotic volume) strengthens our hypothesis that a threshold amount of AQP7 expression is sufficient to maintain the adipocyte features.

Another interesting observation was that AQP7 seems to be relevant not just for glycerol but also for water transport. This study presents, for the first time, a negative correlation between AQP7 water permeability and adipocyte non-osmotic volume and triglyceride content, reinforcing the observation that AQP7 depleted cells are more prone to lipid accumulation. Furthermore, the observed positive correlation between *P*_gly_ and *P*_f_ also discloses the role of AQP7 as both a glycerol and a water channel. The role of AQP7 as a water channel in the context of adipose tissue has been, so far, underrated.

A possible limitation of our calculations for *P*_gly_ and *P*_f_ is the assumption of spherical rather than disc-like shape of the adherent cells. This would be reflected in an overestimation of both permeabilities but with no consequence in their relative values and thus not affecting our previous conclusions. Moreover, the calculated *P*_f_ and *P*_gly_ values are within the range published by [4] in mice adipocytes.

Osmotic stress poses one of the most fundamental challenges to living cells meaning that in order to regain homeostasis cells have to cope rapidly with the environmental perturbations. Aquaporins are among the many mechanisms developed by living organisms to withstand these stresses [19]. However, in the context of obesity, the accumulation of lipid storage and the consequent cell swelling could generate an internal hydrostatic pressure inducing membrane surface tension that could account for AQP7 down-regulation. This aquaporin regulation mechanism has been previously reported for different aquaporins in other systems [20], [21], [22].

In sum, the data presented in the current work indicate that AQP7 controls non-osmotic volume in adipocytes since AQP7 silencing causes enhanced non-osmotic adipocyte volume and triglyceride content. As a complement to the AQP7 expression studies within the obesity and diabetes backgrounds, it would be relevant to further investigate the mechanisms regulating AQP7 activity. Performing functional studies with hypertrophic adipocytes would be relevant to clarify some of these questions. Additionally, it would also be pertinent to investigate if cells trigger any compensatory mechanism capable of sustaining the glycerol fluxes in the absence of AQP7 channels.

## Supporting Information

Figure S1 Analysis of RT-PCR products by agarose gel electrophoresis. PCR products were amplified from white adipose tissue cDNA (5 ng) using different concentrations of eEF2 sense and antisense primers. Lane 1, marker (100 bp DNA Ladder, Genecraft); Lane 2, negative control (no cDNA); Lane 3, 1000 nM of eEF2 primers; *Lane 4*, 100 nM of eEF2 primers; *Lane 5*, 50 nM of eEF2 primers.
(PDF)

Table S1 List of primers used for the study of adipocyte markers expression in adipocytes and stromal vascular fraction (SVF) from white adipose tissue and the relative expression levels.
(PDF)

Table S2 List of primers for the study of SOX9 expression in adipocytes and stromal vascular fraction (SVF) from white adipose tissue and the relative expression levels.
(PDF)
