# Supplementary material for: Biophysical Assessment of Human Aquaporin-7 as a Water and Glycerol Channel in 3T3-L1 Adipocytes
Source: PLoS One. 2013 Dec 20;8(12):e83442. doi: 10.1371/journal.pone.0083442 (PMC3869813; doi:10.1371/journal.pone.0083442)
Supplement: Figure S1 — Analysis of RT-PCR products by agarose gel electrophoresis. PCR products were amplified from white adipose tissue cDNA (5 ng) using different concentrations of eEF2 sense and antisense primers. Lane 1, marker (100 bp DNA Ladder, Genecraft); Lane 2, negative control (no cDNA); Lane 3, 1000 nM of eEF2 primers; Lane 4, 100 nM of eEF2 primers; Lane 5, 50 nM of eEF2 primers. (PDF) [file pone.0083442.s001.pdf]

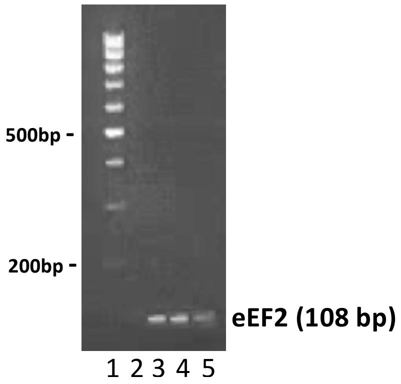

Figure S1 – Analysis of RT-PCR products by agarose gel electrophoresis. PCR products were amplified from white adipose tissue cDNA (5ng) using different concentrations of eEF2 sense and antisense primers. *Lane 1*, marker (100 bp DNA Ladder, Genecraft); *Lane 2*, negative control (no cDNA); *Lane 3*, 1000nM of eEF2 primers; *Lane 4*, 100nM of eEF2 primers; *Lane 5*, 50 nM of eEF2 primers.
