# Supplementary material for: Biophysical Assessment of Human Aquaporin-7 as a Water and Glycerol Channel in 3T3-L1 Adipocytes
Source: PLoS One. 2013 Dec 20;8(12):e83442. doi: 10.1371/journal.pone.0083442 (PMC3869813; doi:10.1371/journal.pone.0083442)
Supplement: Table S1 — List of primers used for the study of adipocyte markers expression in adipocytes and stromal vascular fraction (SVF) from white adipose tissue and the relative expression levels. (PDF) [file pone.0083442.s002.pdf]

Table S1 – List of primers used for the study of adipocyte markers expression in adipocytes and stromal vascular fraction (SVF) from white adipose tissue and the relative expression levels

	Gene name and Reference sequence	Primers sequence (5'→3')	Reference	Relative Expression Level
Adipocyte marker	**aP2** **NM_024406.2**	Sense: TTCGATGAAATCACCGCAGA<br>Antisense: GGTCGACTTTCCATCCCACTT	[1]	Adipocytes 3.90±3.29<br>SVF 0.45±0.26
	**HSL** **NM_001039507.2**	Sense: GGCTTACTGGGCACAGATACCT<br>Antisense: CTGAAGGCTCTGAGTTGCTCAA	[1]	Adipocytes 8.54±4.10<br>SVF 1.09±1.20
	**Perilipin A** **NM_001113471.1**	Sense: TGCTGGATGGAGACCTC<br>Antisense: ACCGGCTCCATGCTCCA	[1]	Adipocytes 7.14±5.49<br>SVF 0.23±0.16
	**GLUT4** **NM_009204.2**	Sense: CTTCGAGACAGCAGGGGTAG<br>Antisense: AGGAGCAGAGCCACAGTCAT	[2]	Adipocytes 5.23±0.23<br>SVF 0.09±0.10
Housekeeping gene	**EeF2** **NM_007907.2**	Sense: GCTTCCCTGTTCACCTCTGACTCTG<br>Antisense: CCGGATGTTGGCTTTCTTGTCC	This work	Adipocytes 1.00±0.62<br>SVF 1.00±0.77

References

1. Gonzalez-Munoz E, Lopez-Iglesias C, Calvo M, Palacin M, Zorzano A, et al. (2009) Caveolin-1 loss of function accelerates glucose transporter 4 and insulin receptor degradation in 3T3-L1 adipocytes. Endocrinology 150: 3493-3502.
2. Bach D, Pich S, Soriano FX, Vega N, Baumgartner B, et al. (2003) Mitofusin-2 determines mitochondrial network architecture and mitochondrial metabolism. A novel regulatory mechanism altered in obesity. J Biol Chem 278: 17190-17197.
