# Supplementary material for: Biophysical Assessment of Human Aquaporin-7 as a Water and Glycerol Channel in 3T3-L1 Adipocytes
Source: PLoS One. 2013 Dec 20;8(12):e83442. doi: 10.1371/journal.pone.0083442 (PMC3869813; doi:10.1371/journal.pone.0083442)
Supplement: Table S2 — List of primers for the study of SOX9 expression in adipocytes and stromal vascular fraction (SVF) from white adipose tissue and the relative expression levels. (PDF) [file pone.0083442.s003.pdf]

Table S2 – List of primers for the study of SOX9 expression in adipocytes and stromal vascular fraction (SVF) from white adipose tissue and the relative expression levels

	Gene name and Reference sequence	Primers sequence (5'→3')	Reference	Relative Expression Level
**Capillary endothelia marker**	**SOX9 NM_011448.4**	Sense: AGGAAGCTGGCAGACCAGTA<br>Antisense: CGTTCTTCACCGACTTCCTC	[Bradley 2010]	Adipocytes 0.14±0.06<br><br>SVF 1.63±0.77
**Housekeeping gene**	**EeF2 NM_007907.2**	Sense: GCTTCCCTGTTCACCTCTGACTCTG<br>Antisense: CCGGATGTTGGCTTTCTTGTCC	This work	Adipocytes 1.00±0.77<br><br>SVF 1.00±0.77

Reference

Bradley EW, Drissi MH (2010) WNT5A regulates chondrocyte differentiation through differential use of the CaN/NFAT and IKK/NF-kappaB pathways. Mol Endocrinol 24: 1581-1593.
